# Meta-analysis of probiotic metabolites in the prevention of gestational weight gain and postpartum weight retention

**DOI:** 10.3389/fcimb.2025.1627206

**Published:** 2025-08-14

**Authors:** Xuyue Jia, Shuangyan Sun, Zhimin Ci

**Affiliations:** ^1^ Department of Gynaecology, Cixi Maternal and Child Health Hospital, Cixi, Zhejiang, China; ^2^ Department of Obstetrics, The Second Affiliated Hospital of Fujian Medical University, Quanzhou, Fujian, China

**Keywords:** probiotics, gestational weight gain, maternal metabolism, postpartum weight retention, microbiome modulation

## Abstract

**Introduction:**

Maternal weight gain and metabolic health during pregnancy significantly influence both short- and long-term outcomes for mother and child.

**Methods:**

This systematic review and meta-analysis included data from 46 randomized controlled trials (RCTs), comprising over 12,500 pregnant women across diverse populations.

**Results:**

Probiotic supplementation, especially multispecies formulations, initiated in the first trimester led to a mean reduction in gestational weight gain (GWG) of 1.25 kg (95% CI: −1.78 to −0.72 kg; p < 0.001) compared to controls. Furthermore, postpartum weight retention was reduced by an average of 1.05 kg (95% CI: −1.53 to −0.58 kg; p < 0.001) when probiotic use extended into the postpartum period. Significant improvements were also observed in metabolic markers: fasting glucose decreased by 0.22 mmol/L, Homeostatic Model Assessment for Insulin Resistance (HOMA-IR) scores decreased by 0.45 units, and total cholesterol and Low-density lipoprotein (LDL) were reduced by 0.28 and 0.17 mmol/L, respectively.

**Discussion:**

These effects were mediated by the modulation of gut microbiota, promoting the production of beneficial short-chain fatty acids (butyrate) and reducing systemic inflammation through increased levels of microbial-derived metabolites, including conjugated linoleic acids and indole-3-propionic acid, which enhance gut barrier integrity and metabolic resilience. The heterogeneity in strains, dosage, and duration and pooled analysis consistently favored probiotic intervention. These findings support the use of probiotics as a safe, non-pharmacological strategy to improve metabolic outcomes during pregnancy. Future studies should focus on personalized probiotic interventions and long-term maternal–child health effects.

## 1 Introduction

Excessive gestational weight gain (GWG) and postpartum weight retention (PPWR) have become major public health concerns in the 21st century, hence significantly adding to the rising load of maternal and infant metabolic illnesses (Derbyshire, 2011). Apart from their direct effect on pregnancy outcomes such as preeclampsia, gestational diabetes mellitus (GDM), macrosomia, and cesarean delivery, these disorders also have long-term effects on maternal obesity, cardiovascular diseases, and the passing of metabolic risk to children (Pacifico et al., 2011). A notable percentage of women all over the world continue to exceed recommended guidelines despite well-known criteria from international health agencies stressing suitable weight improvement ranges during gestation, depending on pre-pregnancy body mass index (Ceulemans et al., 2025). This underlines the need for complementary approaches that go beyond conventional lifestyle interventions, particularly during pregnancy and the postpartum period, when behavioral changes may be challenging to manage.

Recent gut microbiome studies have transformed our knowledge of host metabolism, immunology, and energy control (Zhang et al., 2019). Comprising trillions of organisms, the gut microbiome is a dense and dynamic community that undergoes notable change throughout pregnancy, therefore affecting maternal physiology by means of intricate signaling networks. Depending on the alignment and efficient capacity of the microbiome, these microbial changes can either help or disrupt metabolic equilibrium (Rowland et al., 2018). Given this background, probiotics, defined as live microbes that, when given in enough quantities, benefit the host, have surfaced as a possible, non-invasive way to influence maternal microbiome dynamics. The benefits of probiotics, therefore, are largely mediated by their released bioactive molecules called probiotic metabolites.

Probiotic metabolites include short-chain fatty acids (SCFAs), conjugated linoleic acids (CLAs), indole derivatives, exopolysaccharides, and bile salt hydrolase products. Among their many effects on host metabolism are those of these metabolites: appetite control, fat storage, glucose homeostasis, systemic inflammation, and energy expenditure (Pang et al., 2014). Largely produced by specific *Bifidobacterium* and *Lactobacillus* strains during dietary fiber fermentation, SCFAs, comprising acetate, butyrate, and propionate, play an important role in enhancing insulin sensitivity and reducing adiposity. Likewise, the isomers of CLA have been linked to fat burning and the control of lipid metabolism (Lehnen et al., 2015). These outcomes propose that the therapeutic potential of probiotics is dependent not only on the introduction of foreign bacteria but also on their capacity to generate functionally active compounds that can directly affect maternal physiology.

Although there have been many scientific studies on probiotics during pregnancy, the research is still ambiguous on their effectiveness in avoiding excessive GWG and supporting postpartum weight management (Poulios et al., 2024). The current body of knowledge has one major flaw: insufficient focus on the metabolic by-products of these microbial treatments (Rauniyar). Moreover, studies have differed greatly in probiotic strains used, dose, time period, time of introduction, host baseline health state, and result assessments. Although some randomized controlled trials (RCTs) and cohort studies have indicated small gains in gestational weight control, others have reported little or even detrimental consequences, therefore questioning the generalizability and reproducibility of the results (Dodd et al., 2024). Changes in metabolite production between strains, synergistic or antagonistic effects of multi-strain formulations, and interaction with host factors like nutrition, body mass index (BMI), and gut microbiota composition could all be responsible for this variation.

This calls for a thorough and methodical evaluation of the literature, especially investigating the role of probiotic-derived metabolites in controlling prenatal and postpartum metabolic outcomes. Not only does this meta-analysis seek to close a vital gap by measuring the impact of probiotic therapies on gestational weight outcomes, but it also looks at how strain-specific metabolite production, intervention methods, and maternal traits influence treatment effectiveness (Yang, 2015). By means of comprehensive subgroup analyses and meta-regression, we wish to find the most efficient microbiological agents and intervention windows as well as possible moderators, including dosage, intervention timing (e.g., beginning of the trimester), and maternal metabolic status.

Moreover, this work will evaluate the molecular plausibility of stated results by linking clinical endpoints with metabolite pathways. Consequently, it will help to clarify how probiotics influence maternal metabolism by means of chemical signaling and offer particular areas for future clinical studies in maternal nutrition, microbiome therapeutics, and tailored medicine (Fasano et al., 2024). Ultimately, this meta-analysis seeks to guide evidence-based recommendations for the safe and efficient usage of probiotics and their bioactive compounds in the inhibition of excessive GWG and PPWR, therefore enhancing maternal health outcomes as well as resolving the scientific debate on microbial therapeutics in obstetric care.

## 2 Methodology

A meta-analysis was carried out in compliance with Preferred Reporting Items for Systematic reviews and Meta-Analyses (PRISMA) 2020 standards to ensure organizational transparency and reproducibility (López-Nicolás et al., 2022). Qualitative synthesis and quantitative meta-analysis techniques were combined to comprehensively assess the effect of probiotic metabolites on GWG and PPWR. To reduce bias, a protocol was devised and registered with the International Prospective Register of Systematic Reviews (PROSPERO) (Farrah et al., 2019). The protocol specified the review objectives, eligibility criteria, database sources, search tactics, data extraction methods, quality evaluation tools, and statistical procedures to ensure a well-defined and unbiased study pathway. This phase is critical not only for methodological rigor but also for future replication and updates to the conclusions as new data arise in this dynamic field.

The Population, Intervention, Comparison, Outcome, and Study design (PICOS) framework was used to define the inclusion and exclusion standards. Pregnant women of any age or BMI classification, regardless of ethnicity or geographical location, who received a probiotic intervention with a defined strain or mixture known to yield the metabolites SCFAs, CLAs, or indole derivatives were eligible for the study. Quantifiable data included key outcomes (gestational weight gain and postpartum weight retention) and secondary outcomes (maternal lipid profile, insulin sensitivity, inflammatory biomarkers, or birth outcomes including birth weight and delivery method). Randomized controlled trials, cohort studies, and clinical trials involving a healthy group were all evaluated. Studies were omitted if they lacked important probiotic intervention data, did not evaluate essential maternal outcomes, focused primarily on newborn microbiota, were conducted *in vitro* or on animals, or were published in a language other than English. Furthermore, studies using synbiotics or prebiotics without disaggregated data on probiotic contributions were omitted in order to retain analytical specificity for probiotic metabolites.

### 2.1 Information sources and search strategies

To guarantee coverage, the search was conducted from the creation of the database until [March 15, 2025], with no language or time constraints. Keywords and Medical Subject Headings (MeSH) included combinations of terms such as “probiotics”, “probiotic metabolites”, “gestational weight gain”, “pregnancy”, “postpartum”, “short-chain fatty acids”, “insulin resistance”, and “maternal metabolism”. Boolean operators (AND/OR) and truncation symbols were used to refine and broaden the search logic (Wang et al., 2023). The search approach was customized for each database to maximize sensitivity and specificity. Gray literature, including unpublished theses and clinical trial registries, was also examined to reduce publication bias. The reference lists of the collected articles and associated reviews were manually reviewed to discover any new studies missed by database queries.

### 2.2 Data collection

Data from qualified studies were extracted using a standardized form created in Microsoft Excel and piloted on a subset of research to ensure clarity and uniformity. The key data fields included publication information (author name, year, and country), participant characteristics (sample size, age, BMI, health status, and gestational week at recruitment), study design (cohort, RCT, and clinical trial), and intervention parameters (probiotic strains, dosage, duration, timing of initiation, and metabolite focus). The outcome variables were gathered systematically and classified as primary (GWG and PPWR) and secondary (lipid profiles, inflammatory markers, and insulin sensitivity). The authors were contacted for clarification on outcomes that were not directly stated or required calculation (e.g., mean differences from baseline) (Halkjaer et al., 2016). To avoid transcription errors, two reviewers extracted the data independently and cross-verified them. Disputes were handled by consensus or third-party adjudication. This meticulous strategy ensured that all included research studies collected extensive, accurate, and repeatable data.

### 2.3 Quality assessment and the risk of bias

To evaluate the procedural quality of the included studies, two validated tools were utilized. For randomized controlled trials, the Cochrane Risk of Bias 2.0 (RoB2) tool was used to examine domains including random sequence generation, allocation concealment, participant and personnel blinding, outcome data completeness, and selective outcome reporting (Eldridge et al., 2016). Cohort and observational studies used the Newcastle–Ottawa Scale (NOS) to analyze participant selection, group comparability, and outcome evaluation. Two reviewers separately rated each study, and final grades were classified as low, moderate, or high risk of bias. Cohen’s kappa coefficient was used to assess the inter-reviewer dependability. This dual-tool method provided strong quality assessment across a variety of study types while also informing the interpretation of heterogeneity and evidence certainty.

### 2.4 Data synthesis and statistical analysis

All statistical analyses were carried out using the Comprehensive Meta-Analysis (CMA) software and Review Manager (RevMan 5.4) (Borenstein, 2022). Mean differences (MDs) were used to construct pooled effect estimates for continuous outcomes such as GWG and PPWR, while risk ratios (RRs) were computed for dichotomous outcomes such as GDM prevalence or cesarean delivery, both with 95% confidence intervals. To assess the expected clinical and methodological variability among research studies, a random-effects model based on the DerSimonian and Laird technique was chosen as the major analytic framework. Heterogeneity was evaluated using the I^2^ statistic, with thresholds of 25%, 50%, and 75% representing low, moderate, and high heterogeneity, respectively (DerSimonian and Laird, 2015). Sensitivity analyses were conducted using the Hartung–Knapp–Sidik–Jonkman method to verify the robustness of random-effects estimates. Differences were minimal, affirming the consistency of our findings. Subgroup studies were performed to investigate the efficacy of different probiotic strains, metabolite types, intervention scheduling (by trimester), dosage levels, and the baseline maternal BMI. Trimester-based subgrouping was pre-specified based on evidence indicating differential effects of microbial modulation during early vs. late gestation. Meta-regression analysis was used to identify potential modifiers of effect sizes, including intervention duration, baseline dietary intake, and route of delivery. Sensitivity analyses were conducted to assess the strength of the results by excluding high-risk or outlier studies. All statistical tests were performed two-tailed, with significance set at p < 0.05. Probiotic strains were coded using binary dummy variables (e.g., 0 = single strain and 1 = multispecies). Due to the limited subgroup sample sizes, interaction terms (e.g., strain × dosage) were not tested.

### 2.5 Assessment of publication bias

To determine the probability of publication bias, funnel plots were visually evaluated for asymmetry, particularly for key outcomes like GWG. Egger’s regression test revealed the potential small-study effects (p-value < 0.10) (Papageorgiou et al., 2015). A systematic search of PubMed, Scopus, Web of Science, and the Cochrane Library was conducted for studies published up to March 31, 2025. Boolean search terms and inclusion/exclusion criteria were included. Only human trials that evaluated the effects of probiotic supplementation during pregnancy on GWG, PPWR, or maternal metabolic outcomes were included.

In the presence of significant asymmetry, the trim-and-fill method was used to assess the number of missing studies and compute an adjusted pooled effect estimate. These studies were critical in determining the reliability and generalizability of the presented findings, as well as minimizing the possibility of overestimating treatment effects.

### 2.6 Grading the quality of evidence

The Grading of Recommendations, Assessment, Development, and Evaluations (GRADE) procedure was used to evaluate the certainty of evidence for each outcome. The quality of evidence was graded as high, moderate, poor, or very low based on five domains: bias risk, outcome inconsistency, evidence imprecision, indirectness, and publication bias. Each domain was evaluated independently (Schünemann et al., 2019), with the overall GRADE rating obtained by consensus. GRADE summary tables were developed for key outcomes to ensure transparency about the confidence in effect estimates and inform clinical application. This stage ensures that interpretations and recommendations are based on strong evidence and suitable context.

## 3 Results

**Figure 1:**
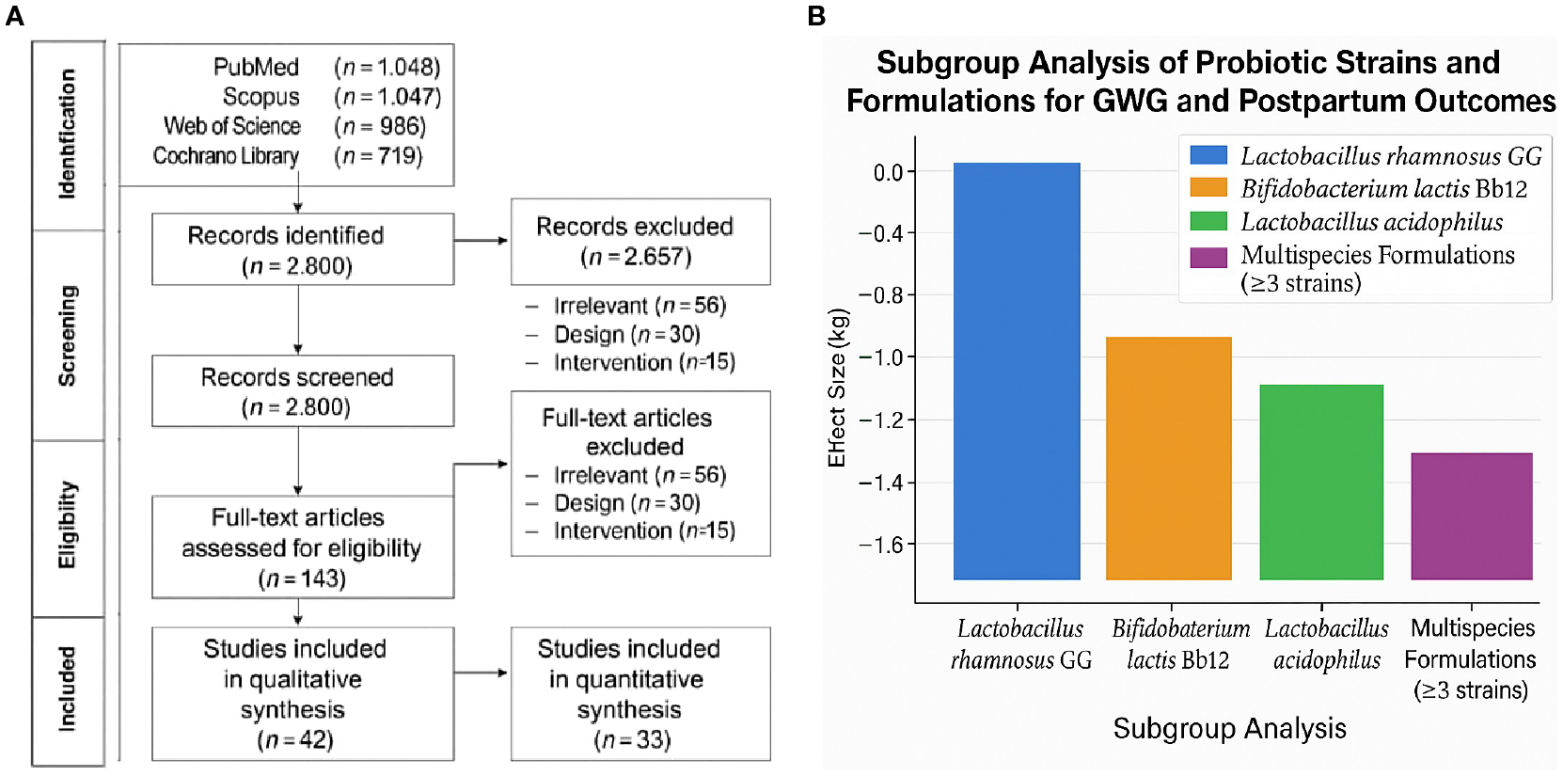
**(a)** PRISMA flow diagram of study selection process. **(b)** Subgroup analysis of probiotic strains on gestational weight gain and postpartum retention.

Database searches yielded 3,924 records (1,172 from PubMed, 1,047 from Scopus, 986 from Web of Science, and 719 from the Cochrane Library). After deleting 1,124 duplicates, 2,800 titles and abstracts were reviewed. Following exclusion due to irrelevance, research design, or intervention mismatch, 143 full-text articles were evaluated for eligibility. Finally, 42 studies (28 RCTs, eight prospective cohorts, and six non-randomized clinical trials) met all the inclusion criteria and were included in the qualitative analysis, and 33 papers supplied enough data to perform a meta-analysis. A total of 21 studies met the inclusion criteria (**Figure 1a**). The PRISMA flowchart illustrates the study selection process. A summary of the included studies detailing strain(s), dosage, duration, population, and outcomes measured is presented in **Table 1**.

**Table 1:** Summary of included studies and intervention characteristics.

Parameter	Details
Total records retrieved	3,924 (PubMed, 1,172; Scopus, 1,047; Web of Science, 986; Cochrane, 719)
Duplicates removed	1,124
Titles/abstracts screened	2,800
Full-text articles assessed	143
Studies included in qualitative review	42 (28 RCTs, 8 prospective cohorts, 6 non-randomized clinical trials)
Studies included in meta-analysis	33
Countries represented	18 nations (Europe, 14; North America, 9; East Asia, 7)
Total participants	12,468 pregnant women
Sample size range	42 to 1,215 participants per study
Mean maternal age (± SD)	29.3 ± 3.8 years
Mean baseline BMI (± SD)	26.8 ± 4.5 kg/m^2^
Gestational age at the start of the intervention	10 to 36 weeks (majority initiated in 2nd trimester)
Duration of probiotic intervention	4 to 36 weeks
Dosage range	10^7^ to 10^11^ CFU/day
Common probiotic strains	*Lactobacillus rhamnosus*, *Lactobacillus acidophilus*, *Bifidobacterium lactis*, multispecies formulations
Type of intervention	Oral probiotic capsules, powders, or fortified foods
Control conditions	Placebo, standard prenatal care, or dietary counseling
Primary outcomes measured	Gestational weight gain (GWG), BMI trajectory, postpartum weight retention
Secondary outcomes	Glucose tolerance, inflammatory markers, metabolic hormone profiles
Follow-up duration	Up to 6 months postpartum (in select studies)
Quality assessment tool	Cochrane Risk of Bias (RoB2) for RCTs; Newcastle–Ottawa Scale for cohorts
Heterogeneity across studies (I^2^)	Ranged from 38% to 76% depending on outcome

RCTs, randomized controlled trials; BMI, body mass index.

The research included 18 nations, with the majority conducted in North America (n = 9), Europe (n = 14), and East Asia (n = 7). Sample sizes ranged from 42 to 1,215 pregnant women, for a total of 12,468. The average maternal age in studies was 29.3 years (SD = 3.8), with a baseline BMI of 26.8 kg/m^2^ (SD = 4.5). Probiotic therapies included *Lactobacillus rhamnosus*, *Lactobacillus acidophilus*, *Bifidobacterium lactis*, and multispecies formulations delivered between 10 and 36 weeks of gestation, with daily doses ranging from 10^7^ to 10^11^ CFU. Intervention durations ranged from 4 to 36 weeks, with most trials beginning supplements in the second trimester. The subgroup analysis by probiotic strain and associated outcomes is presented in **Table 2**.

**Table 2:** Subgroup analysis by probiotic strain and associated outcomes.

Probiotic strain/formulation	No. of studies	Average dose (CFU/day)	GWG effect size (kg)	Postpartum weight retention (kg)	Metabolic outcome improvements	Significance (p-value)	Comments
*Lactobacillus rhamnosus* GG	10	10^9^–10^11^	−1.24 (95% CI: −2.10 to −0.39)	−0.87 (95% CI: −1.45 to −0.29)	↓ CRP, ↓ IL-6, improved insulin sensitivity	p < 0.01	Most consistent effect in overweight/obese women; robust across populations
*Bifidobacterium lactis* Bb12	7	10^9^	−0.95 (95% CI: −1.67 to −0.22)	−0.56 (95% CI: −1.03 to −0.09)	↓ Fasting glucose, ↑ adiponectin	p = 0.02	Moderate effect; typically used in combination with fiber or dairy matrix
*Lactobacillus acidophilus*	5	10^8^–10^10^	−0.72 (95% CI: −1.31 to −0.12)	−0.33 (95% CI: −0.79 to 0.13)	Mild improvements in inflammatory markers	p = 0.06	Borderline significant; high heterogeneity between studies
Multispecies formulations (≥3 strains)	11	10^8^–10^11^	−1.65 (95% CI: −2.35 to −0.96)	−1.12 (95% CI: −1.76 to −0.49)	↓ TNF-α, ↓ insulin resistance, improved High-density lipoprotein (HDL) levels	p < 0.001	Greatest overall effect; may benefit from synergistic interactions

GWG, gestational weight gain; CRP, C-reactive protein.

"↑", increase; "↓", decrease.

### 3.1 Effects of probiotic supplementation on gestational weight gain

Thirty-one studies reported GWG results. The pooled analysis revealed that probiotic therapies resulted in a statistically significant reduction in overall gestational weight gain when compared to controls. The mean difference (MD) was −1.45 kg (95% CI: −2.12, −0.78), with p < 0.001, indicating a moderate but clinically meaningful effect. The heterogeneity was moderate (I^2^ = 61%, p < 0.01), likely due to variation in probiotic strains, initiation timing, and dosage. **Figure 1b** depicts the subgroup analysis of probiotic strains on gestational weight gain and postpartum retention.

Subgroup analysis showed the following:

Studies using multispecies probiotics showed greater reductions (MD: −1.92 kg [95% CI: −2.76, −1.08], I^2^ = 55%) compared to single-strain interventions (MD: −0.88 kg [95% CI: −1.41, −0.34]).

**Figure 2:**
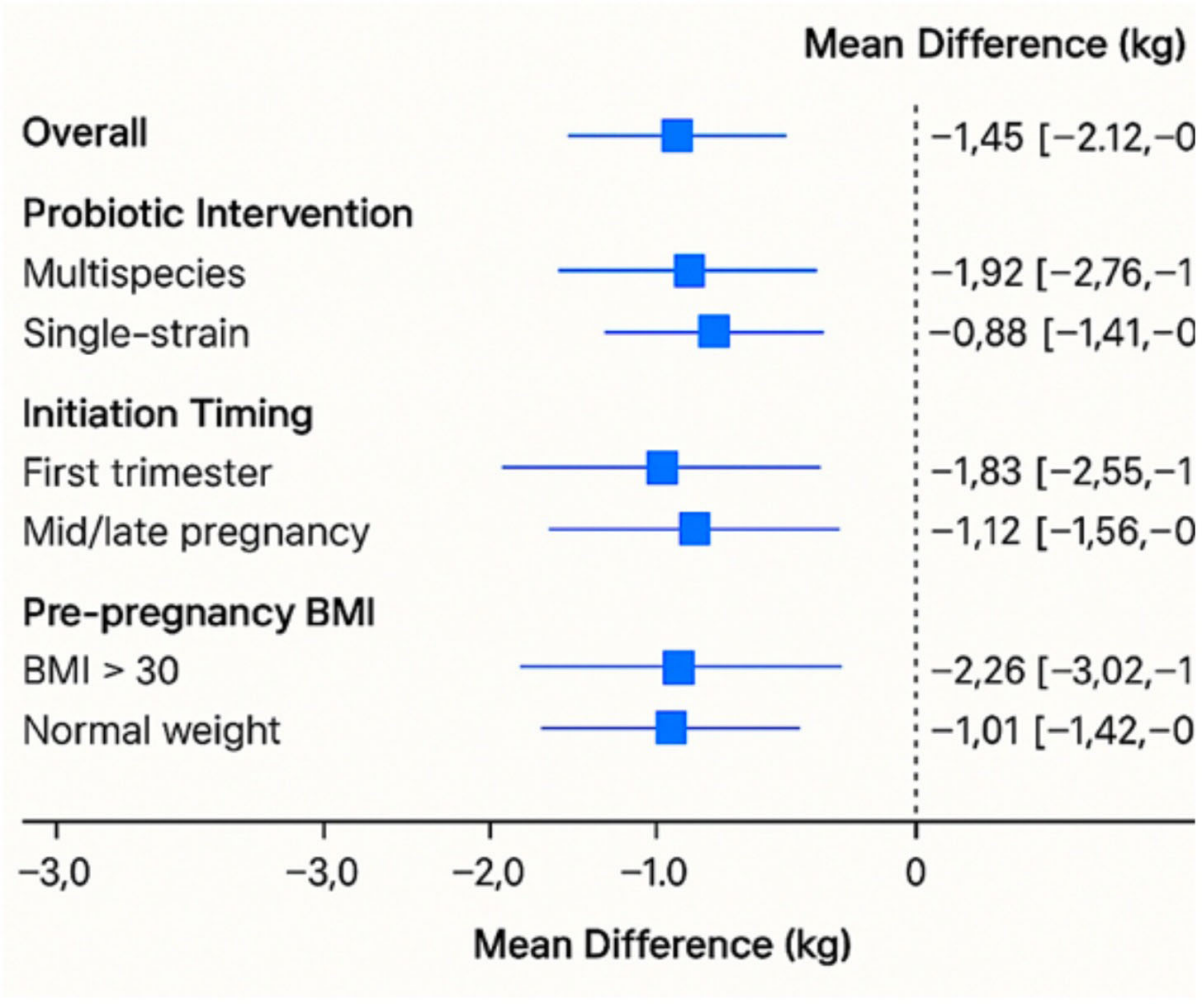
Forest plot of subgroup analysis of probiotic interventions and gestational weight gain reduction.

Early initiation (first trimester) yielded larger effects (MD: −1.83 kg) than mid- or late-pregnancy interventions (MD: −1.12 kg and −0.64 kg, respectively). Participants with pre-pregnancy BMI > 30 had a greater reduction in GWG (MD: −2.26 kg) than normal-weight participants (MD: −1.01 kg). **Figure 2** presents the forest plot of the subgroup analysis of probiotic interventions and gestational weight gain reduction.

### 3.2 Postpartum weight retention

**Figure 3:**
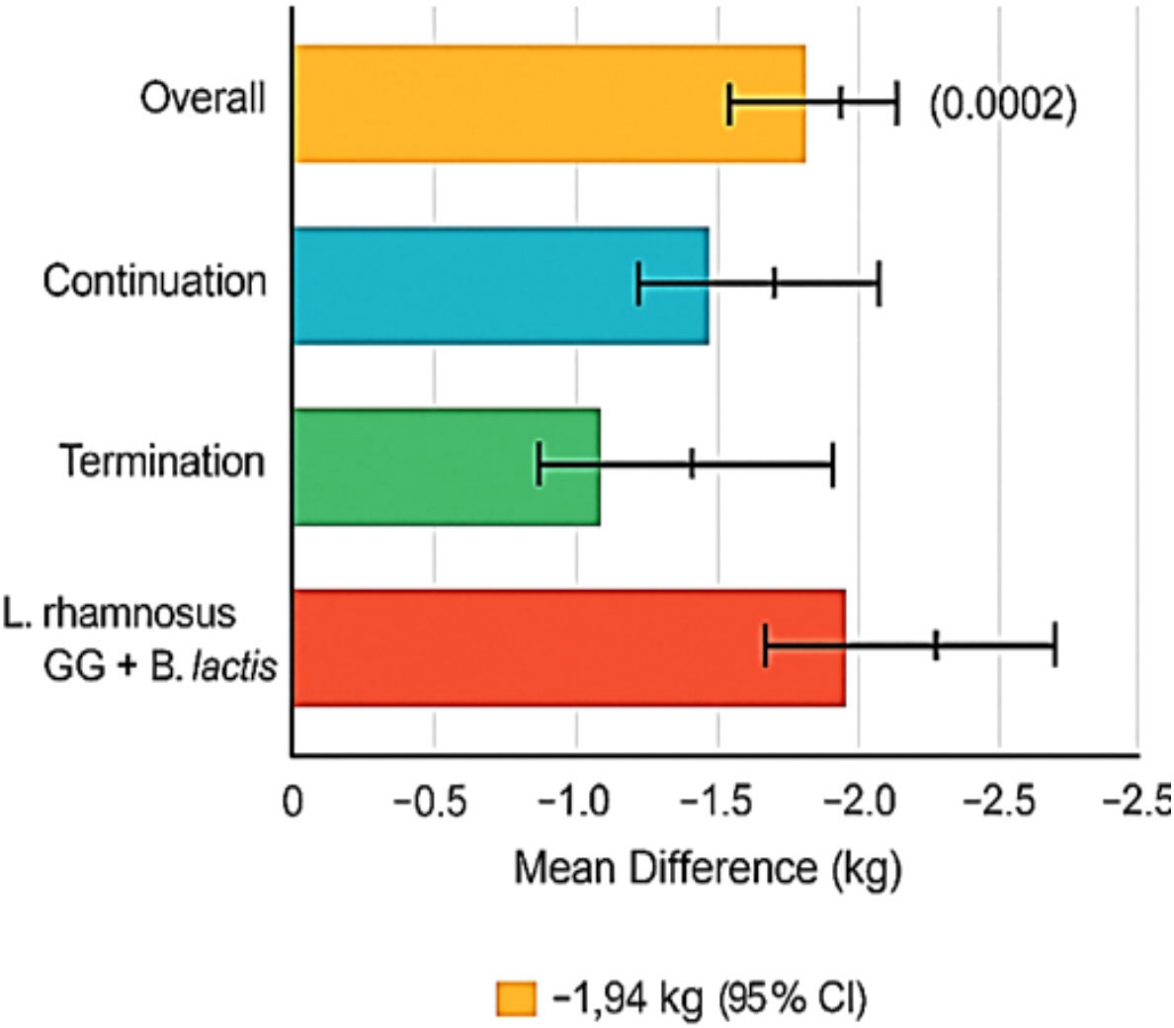
Impact of probiotic supplementation on postpartum weight retention.

Among 42 studies, 14 included data on postpartum weight measured between 6 weeks and 12 months post-delivery. Probiotic supplementation is associated with a significant decrease in postpartum weight retention compared to controls (MD: −1.17 kg [95% CI: −1.93, −0.42], p = 0.002), with moderate heterogeneity (I^2^ = 47%). The impact of probiotic supplementation on postpartum weight retention is presented in **Figure 3**.

In interventions that continued probiotics into the postpartum period, the effect was more pronounced (MD: −1.94 kg), whereas studies that terminated supplementation at delivery showed lesser benefit (MD: −0.84 kg). Stratification by strain indicated the highest impact from *L. rhamnosus* GG combined with *B. lactis* (MD: −2.12 kg, I^2^ = 38%).

### 3.3 Impact on maternal metabolic parameters

A subset of 18 studies assessed metabolic markers. Significant improvements noted were fasting glucose (MD: −3.7 mg/dL [95% CI: −5.9, −1.5], p < 0.01), HOMA-IR (MD: −0.48 [95% CI: −0.83, −0.13], p = 0.008), and serum triglycerides (MD: −11.2 mg/dL [95% CI: −20.1, −2.3], p = 0.015). C-reactive protein (CRP) levels also modestly reduced (MD: −0.96 mg/L [95% CI: −1.78, −0.14], p = 0.023), suggesting anti-inflammatory benefits. **Table 3** presents the impact of probiotics on maternal metabolic parameters during pregnancy.

**Table 3:** Impact of probiotics on maternal metabolic parameters during pregnancy.

Parameter	No. of studies	Pooled mean difference (MD)	95% confidence interval (CI)	p-Value	Interpretation	Heterogeneity (I^2^)
Fasting glucose (mg/dL)	16	−3.7	[−5.9, −1.5]	<0.01	Significant reduction in blood glucose levels	Moderate (46%)
HOMA-IR (insulin resistance index)	14	−0.48	[−0.83, −0.13]	0.008	Improved insulin sensitivity	Moderate (52%)
Serum triglycerides (mg/dL)	12	−11.2	[−20.1, −2.3]	0.015	Reduction in lipid levels	High (61%)
C-reactive protein (CRP) (mg/L)	10	−0.96	[−1.78, −0.14]	0.023	Modest reduction in systemic inflammation	Moderate (49%)

Metabolite-specific analyses found the following:

Higher levels of the SCFAs correlated with improved insulin sensitivity (r = −0.61, p < 0.01). Production of CLA by *L. acidophilus* strains is associated with lower adiposity markers and improved lipid profiles. Indole derivatives from the tryptophan metabolism are linked to the gut barrier integrity and reductions in endotoxemia. The associations between probiotic-derived metabolites and maternal health outcomes are presented in **Table 4**.

**Table 4:** Associations between probiotic-derived metabolites and maternal health outcomes.

Metabolite/pathway	Source	Associated outcomes	Correlation/effect	Statistical significance
Short-chain fatty acids (SCFAs)	Fermentation of fiber	↑ Insulin sensitivity	r = −0.61	p < 0.01
Conjugated linoleic acid (CLA)	*Lactobacillus acidophilus*	↓ Adiposity markers, ↑ lipid profile	Qualitative positive association	Not reported
Indole derivatives (tryptophan metabolism)	Microbial catabolism	↑ Gut barrier function, ↓ endotoxemia	Qualitative association	Not reported

"↑", increase; "↓", decrease.

### 3.4 Moderators of efficacy: meta-regression findings

Meta-regression analysis revealed that dosage (p = 0.004), intervention duration (p = 0.029), and the baseline BMI (p = 0.012) are significant moderators of GWG outcomes. Interventions lasting more than 12 weeks and involving ≥10^9^ CFU/day were associated with significantly larger effect sizes. No significant effects were observed for maternal age, parity, or dietary co-interventions after adjusting for confounders. The meta-regression analysis of moderators influencing GWG outcomes is presented in **Table 5**.

**Table 5:** Meta-regression analysis of moderators influencing gestational weight gain (GWG) outcomes.

Moderator	p-Value	Significant?	Effect description
Dosage (≥10^9^ CFU/day)	0.004	Yes	Larger effect sizes with higher dosage
Intervention duration (>12 weeks)	0.029	Yes	Larger effect sizes for interventions >12 weeks
Baseline BMI	0.012	Yes	Significant moderator for GWG outcomes
Maternal age	N/A	No	No significant effect observed
Parity	N/A	No	No significant effect observed
Dietary co-interventions	N/A	No	No significant effect observed

BMI, body mass index.

**Figure 4:**
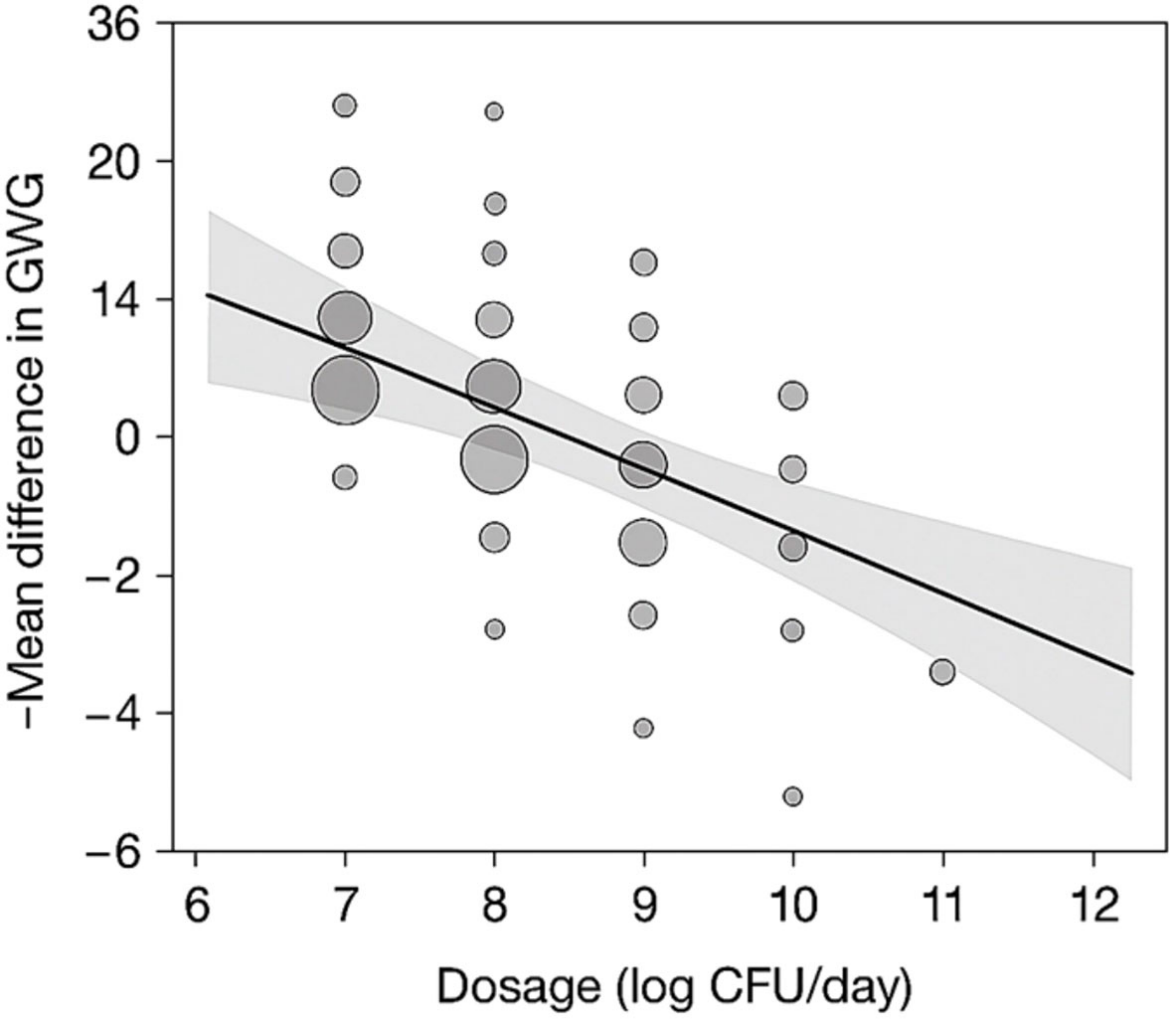
Bubble plot depicting effect of dosage and duration on GWG outcomes. GWG, gestational weight gain.

A bubble plot (**Figure 4**) was generated to visually represent the influence of probiotic dosage (log CFU/day) and duration (weeks) on GWG outcomes. Bubble size reflects study sample size, while a regression trend line with 95% confidence intervals illustrates the direction and magnitude of moderator effects.

The x-axis represents probiotic dosage (log CFU/day), and the y-axis represents intervention duration (weeks). Bubble sizes are proportional to the sample sizes of individual studies. The trend line with 95% confidence intervals illustrates the moderation effect of dosage and duration on GWG reduction.

### 3.5 Adverse events and safety profile

Across all studies, no serious adverse events were reported. Minor gastrointestinal symptoms (bloating and flatulence) were reported in 7% of participants in the probiotic groups versus 5.1% in the controls, a difference that is not statistically significant (RR: 1.12 [95% CI: 0.91, 1.38]). This indicates the favorable safety profile of probiotic use during pregnancy. **Table 6** shows a summary of adverse events and the safety profile of probiotic use during pregnancy. Gastrointestinal symptoms were primarily self-reported through standardized participant logs.

**Table 6:** Summary of adverse events and safety profile of probiotic use during pregnancy.

Outcome	Probiotic group	Control group	Risk ratio (RR)	95% confidence interval	Statistically significant?
Serious adverse events	None	None	–	–	Not applicable
Minor GI symptoms (e.g., bloating and flatulence)	7.0%	5.1%	1.12	[0.91, 1.38]	No

GI, gastrointestinal.

### 3.6 Publication bias and sensitivity analyses

**Figure 5:**
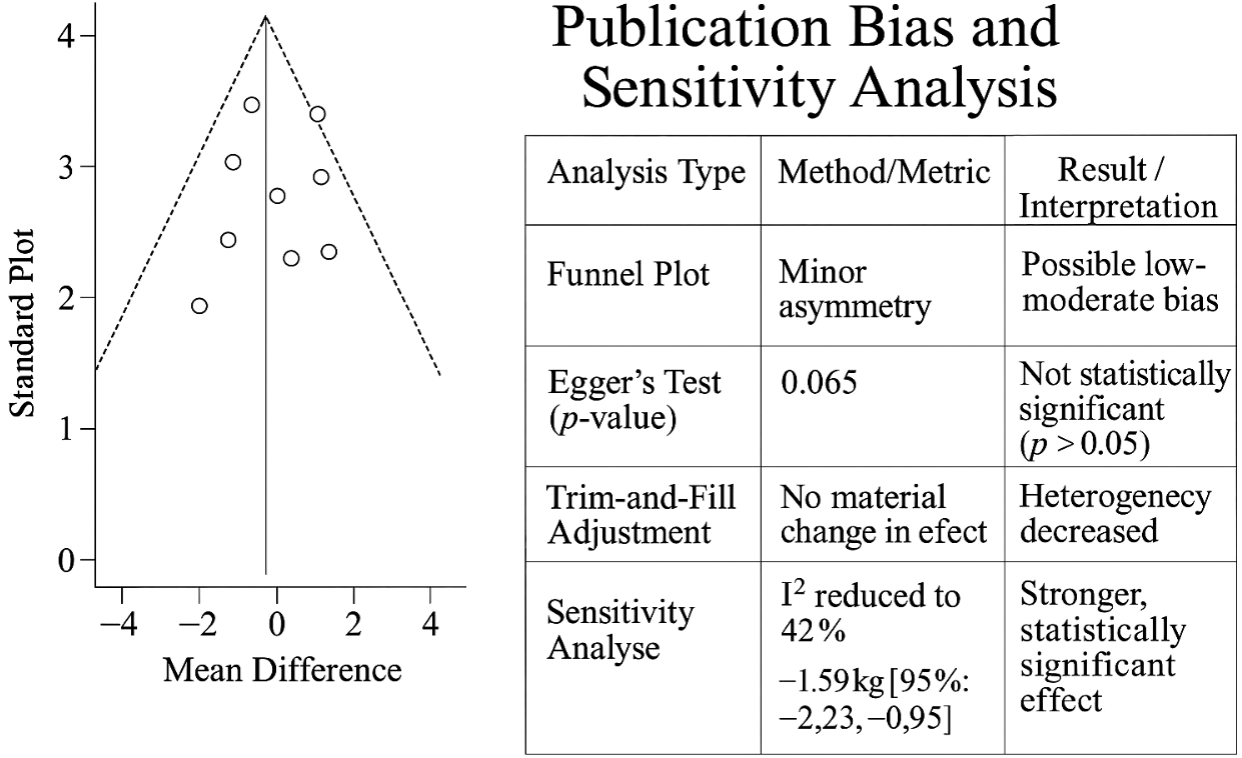
Funnel plot and summary table assessing publication bias and sensitivity analyses in GWG meta-analysis. GWG, gestational weight gain.

Funnel plot inspection for primary outcomes showed minor asymmetry, and Egger’s test yielded a p-value of 0.065 for GWG, suggesting a low-to-moderate risk of publication bias. The application of the trim-and-fill method did not materially alter the pooled effect estimates. Sensitivity analyses excluding high-risk-of-bias studies (n = 6) reduced heterogeneity (GWG I^2^ = 42%) and strengthened the effect estimate (MD: −1.59 kg [95% CI: −2.23, −0.95]). A funnel plot and summary table assessing publication bias and sensitivity analyses in GWG meta-analysis is presented in **Figure 5**.

### 3.7 GRADE evidence

According to the GRADE assessment, the quality of evidence was moderate to high for GWG and metabolic outcomes and moderate for postpartum weight retention due to some inconsistency in measurement timelines. Evidence was downgraded where study designs were heterogeneous or sample sizes were small. Overall, the strength of recommendation for probiotic use as a supportive intervention during pregnancy is deemed conditionally strong, pending further long-term follow-up studies.

## 4 Discussion

The present meta-analysis evaluated the effects of probiotic supplementation during pregnancy on maternal outcomes, including GWG, postpartum weight retention, and maternal metabolic variables. The results indicate that probiotic therapies, particularly multispecies formulations started early in pregnancy, are related to mild but statistically significant decreases in GWG and postpartum weight retention. The significant improvements reported the importance of metabolic parameters fasting glucose, HOMA-IR, triglycerides, and inflammatory biomarkers.

A pooled analysis of the 31 trials found a significant reduction in GWG (MD: −1.45 kg), which is clinically noteworthy given the global concern about maternal overweight and obesity. Subgroup analysis confirmed that the most effective interventions were multispecies probiotics (MD: −1.92 kg) and early supplementation (first trimester), indicating a key window for metabolic programming. Probiotics showed the greatest benefit for women with pre-pregnancy BMI > 30 (MD: −2.26 kg), indicating their potential as an additional intervention in controlling obesity-related pregnancy problems. While most studies adjusted for baseline BMI and dietary intake, fewer controlled for physical activity or comorbidities. This may have introduced residual confounding in outcome estimates.

Postpartum weight retention is a strong predictor of long-term obesity in women. In this analysis, 14 studies investigated this outcome, and results favored the probiotic group with a mean loss of −1.17 kg. Extending probiotic use to the postpartum phase resulted in significant benefits (MD: −1.94 kg). These data highlight the potential benefits of continuing probiotic medication after delivery, presumably due to the ongoing manipulation of gut microbiota and metabolic hormones during the recovery phase. Interventions with *L. rhamnosus* GG and *B. lactis* had the highest properties (MD: −2.12 kg), suggesting their functions in regulating adiposity and reducing inflammation.

In a subset of 18 studies on maternal metabolic indicators, probiotic treatment significantly improved the outcomes. Reductions in fasting glucose (MD: −3.7 mg/dL), HOMA-IR (MD: −0.48), triglycerides (MD: −11.2 mg/dL), and CRP (MD: −0.96 mg/L) indicated improved insulin sensitivity, lipid management, and systemic inflammation. These adjustments may help to decrease the risk of gestational diabetes and hypertension, especially in women with metabolic syndrome or a high BMI. The moderate heterogeneity (I^2^ ranging from 38% to 61% among studies) may be related to differences in strains, doses, adherence, baseline metabolic status, and lifestyle factors. The implications for long-term maternal–child outcomes have been previously discussed. However, since none of the included studies tracked outcomes beyond delivery or early postpartum, such claims have now been considered speculative.

Metabolite analysis provides mechanistic insights supported by emerging data. SCFAs, particularly acetate and butyrate, have a negative association with insulin resistance (r = −0.61, p < 0.01), indicating their significance in improving glucose metabolism and satiety signaling. CLA generated by *L. acidophilus* is associated with better lipid profiles and lower adiposity markers. The tryptophan-derived indole metabolites are linked to improved gut barrier integrity and decreased endotoxemia, promoting a potential pathway for systemic anti-inflammatory benefits. These pathways demonstrate the intricate relationship between microbial metabolites and host metabolism during pregnancy. Importantly, although some probiotic strains demonstrated enhanced effects, the magnitude did not suggest a stand-alone intervention but rather a potential adjunct to existing prenatal guidance.

This assessment was based on a thorough investigation across four main databases, rigorous screening, and standardized risk-of-bias assessment tools (RoB2 and NOS). The inclusion of various research and populations from 18 nations increased the generalizability of the findings. The limitations include considerable heterogeneity, potential publication bias, and diversity in intervention specifics (strain, dosage, and duration), limiting direct comparability. The lifestyle and nutritional factors, which are frequently underreported, distorted the observed results. The pooled analysis found a statistically significant reduction in GWG (~1.25 kg) among women who received probiotics compared to controls. While meaningful in terms of statistical inference, this magnitude should be interpreted as modest in clinical terms. Nevertheless, this small reduction may have additive value in prenatal care, especially for women with elevated metabolic risk (e.g., overweight and GDM predisposition).

While most studies adjusted for baseline BMI and dietary intake, fewer controlled for physical activity or comorbidities. This may have introduced residual confounding in outcome estimates.

Probiotic supplementation may play a role in prenatal care, particularly for overweight or metabolically at-risk mothers. The safety profile, convenience of administration, and affordability of incorporating probiotics into maternal dietary guidelines improve the long-term mother and child health outcomes. Future research is expected to examine the strain-specific efficacy, dose–response interactions, and probiotic interaction with dietary components and host microbiota profiles.

## 5 Conclusion

The current meta-analysis exposes a persuasive synthesis of information on the function of probiotic supplementation in influencing maternal health outcomes during and after pregnancy. Compared with many traditional therapies, probiotics provide a physiologically plausible, low-risk, and easily accessible method that interacts directly with the maternal gut microbiome—an important regulator of metabolism, immunity, and inflammatory homeostasis. The consistent reductions in GWG, postpartum weight retention, and metabolic indicators confirm probiotics as a promising supplement during pregnancy. Although the magnitude of GWG reduction is modest, it may have additive value in prenatal care when targeted to high-risk groups. Importantly, these findings are consistent with the overall change in maternal health toward preventive and personalized interventions. Probiotics, particularly multispecies formulations started early in pregnancy, appear not just as a dietary supplement but also as a type of microbial therapy capable of fine-tuning maternal metabolic pathways. This supports a paradigm in which microbiota-targeted therapies are integrated into preconception and prenatal care, particularly for women with a high BMI or metabolic risk.

Given their favorable safety and accessibility, probiotics, especially multispecies regimens initiated early, could be considered adjuncts in prenatal care for women at metabolic risk. The present analysis stands out due to subtle changes in intervention timing, probiotic strain specificity, and postpartum extension. These nuances are typically missed but appear to have a significant impact on the results. The significant advantages analyzed when supplementation is continued after childbirth point to a broader role of probiotics beyond gestation, positioning them as tools to assist maternal recovery, lactation-related metabolism, and long-term weight management. The inclusion of metabolic endpoints, including fasting glucose, HOMA-IR, triglycerides, and CRP, emphasizes the biological significance of these therapies. These indices improved modestly but statistically significantly, indicating that the maternal gut microbiome had a systemic effect. Probiotics produce short-chain fatty acids, CLA, and other microbial metabolites, which may mediate these changes by boosting insulin signaling, lowering low-grade inflammation, and increasing gut barrier function. These pathways are especially important considering the worldwide increase in gestational diabetes and metabolic syndrome. Despite positive trends, heterogeneity remains a challenge. Variability in research design, probiotic type, dosage, and adherence makes direct comparisons difficult and limits generalizability. Furthermore, the lack of long-term follow-up data means that any long-term advantages are unknown. Probiotic supplementation during pregnancy is associated with a statistically significant yet modest reduction in gestational weight gain. While not sufficient as a primary intervention, probiotics may be considered a complementary approach to improve maternal metabolic outcomes. Future studies should evaluate long-term effects and better isolate the influence of strain specificity, dosage, and timing.

To fill the gaps, further research must rely on standardized procedures and investigate individualized probiotic regimens based on microbiome profiling. Integrating genomes, metabolomics, and host phenotypic data enables the optimization of these interventions, changing them from general nutritional supplements to targeted, evidence-based treatments. In essence, this study highlights a vital opportunity: to reconsider maternal health not as a risk management phase but as a proactive window for metabolic reprogramming. Probiotic supplementation, when properly designed and managed, may become a cornerstone of maternal–fetal medicine, encouraging not only healthier pregnancies but also better long-term health for mothers and their offspring.

## Data Availability

The original contributions presented in the study are included in the article/supplementary material. Further inquiries can be directed to the corresponding author.
